# Molecular Characterization of Fructose-1,6-bisphosphate Aldolase From *Trichinella spiralis* and Its Potential in Inducing Immune Protection

**DOI:** 10.3389/fcimb.2019.00122

**Published:** 2019-04-24

**Authors:** Yong Yang, Xue Bai, Chengyao Li, Mingwei Tong, Peihao Zhang, Wei Cai, Xiaolei Liu, Mingyuan Liu

**Affiliations:** ^1^Key Laboratory of Zoonosis Research, Ministry of Education, College of Veterinary Medicine, Institute of Zoonosis, Jilin University, Changchun, China; ^2^Wu Xi Medical School, Jiangnan University, Wuxi, China; ^3^School of Basic Medical Sciences, Shanxi Medical University, Taiyuan, China; ^4^Affiliated Hospital of Jiangnan University, The Fourth People's Hospital of Wuxi City, Wuxi, China; ^5^Jiangsu Co-innovation Center for Prevention and Control of Important Animal Infectious Diseases and Zoonoses, Yangzhou, China

**Keywords:** food-borne parasites, *Trichinella spiralis*, fructose-1, 6-bisphosphate aldolase, immune protection, vaccine

## Abstract

*Trichinella spiralis* is a major food-borne parasite worldwide. Trichinellosis caused by *T. spiralis* is not only a public health problem, but also an economic hazard in food safety. The development of effective vaccines to prevent *Trichinella* infection in domestic animals and humans is urgently needed for controlling of this zoonosis. Fructose-1, 6-bisphosphate aldolase (FBPA) is involved in energy production in glycolysis and is also associated with many non-glycolysis functions in the parasite, such as adhesion to host cells, plasminogen binding, and invasion. FBPA has been considered as a potential vaccine candidate or as a target for chemotherapeutic treatment. Here, we report for the first time the characterization of FBPA of *T. spiralis* and an evaluation of its potential as a vaccine candidate antigen against *T. spiralis* infection in mice. The results of qPCR and western blot analysis showed that the *Ts*-FBPA gene was expressed at various developmental stages of *T. spiralis* and was also detected in excretory–secretory products (ES) of *T. spiralis* muscle larvae (ML). Immunostaining with anti-*Ts*-FBPA mouse sera indicated that it localized principally to the surface and embryos of this parasitic nematode. Vaccination of mice with recombinant *Ts*-FBPA (r*Ts*-FBPA) resulted in a Th1/Th2 mixed humoral and cellular immune response with Th2 predominant, as well as remarkably elevated IgE levels. Moreover, mice vaccinated with r*Ts*-FBPA displayed a 48.7% reduction in adult worm burden and 52.5% reduction in muscle larval burden. These studies indicated that *Ts*-FBPA is a promising target for developing an effective vaccine to prevent and control *Trichinella* infection.

## Introduction

*Trichinella spiralis* (*T. spiralis)* can infect a wide variety of mammalian animals including humans, and it is one of the most widespread food-borne parasites throughout the world. Accidentally digestion of raw or undercooked meat containing infectious larvae of *T. spiralis* results in human infections. Owing to alteration of diet and cooking habits and an increase in meat demand, trichinellosis is considered as an emerging or re-emerging infectious disease (Gottstein et al., 2009). Human trichinellosis has been found in 66 countries and pork is the main source of occurrence of human *Trichinella* infection in China (Cui et al., 2011; Jiang et al., 2016). Trichinellosis is hazardous to both the public health and the economic productivity in pig industry (Cui and Wang, 2011). On account of extensive distribution of domesticated and wild animal reservoirs, as well as the sources of human trichinellosis, this foodborne parasitosis is hard to prevent and control (Bai et al., 2017). Antihelminthics are widely used to control this parasite. However, the abuse of these chemicals resulted in the emergence of chemical residues in meat, environmental pollution, and resistant forms of the parasite. Therefore, the development of effective vaccines against *Trichinella* infection in pigs and humans is a promising measures to control trichinellosis (Bai et al., 2017; Zhang et al., 2018).

In the last decade, several proteins involved in host invasion, parasite survival, and immunity have been developed as vaccine candidates and their protective effect against *Trichinella* larvae challenge in animal models has been investigated (Feng et al., 2013; Gu et al., 2017; Song et al., 2018; Yang et al., 2018). Some of these vaccine candidates offered significant protection against infection, but currently there is no vaccine that provides adequate protection against *Trichinella* infection for use commercially. More work is required for identification of vaccine candidates which can induce stronger protective immune responses against *Trichinella* infection.

Fructose-1,6-bisphosphate aldolase (FBPA) is an important enzyme in glycometabolism. FBPA can hydrolyse fructose 1,6-bisphosphate to glyceraldehyde 3-phosphate (GAP) and dihydroxyacetone phosphate (DHAP). FBPA can be classified into two subtypes according to its catalytic mechanisms. Class-I enzymes are mainly expressed in animals and plants, while Class-II enzymes are found in bacteria and lower eukaryotes (Maurady et al., 2002). FBPA has been identified in many parasites and plays an essential role during the development and survival of helminths, is involved in nutrient transport through the tegument, egg laying and muscular activity due to its involvement in carbohydrate metabolism (Lorenzatto et al., 2012; Li et al., 2014; Hu et al., 2015). FBPA not only contributes to energy generation in the glycolytic pathway but also has non-glycolytic effects on various key processes, for instance, adhesion to host cells, plasminogen binding, invasion, and immune evasion (Starnes et al., 2009; Lorenzatto et al., 2012; Hu et al., 2015). As FBPA plays a central role in parasite activities and survival it has been considered as a potential vaccine candidate or as a chemotherapeutic target for treatment. Several reports have also indicated the protective efficacy of FBPA against various parasite challenge (El-Dabaa et al., 1998; McCarthy et al., 2002; Marques et al., 2008). In the current study, we cloned and characterized the FBPA gene of *T.spiralis* (*Ts*-FBPA). The expression patterns in the developmental stages of *Trichinella* and the immune protection against *Trichinella* infection in mice induced by recombinant *Ts-*FBPA (r*Ts*-FBPA) were also investigated.

## Materials and Methods

### Parasites and Antigens Preparation

*T. spiralis* (ISS534) parasites were maintained by serial passage in female ICR mice. Muscle larvae (ML) of *T. spiralis* were recovered from infected ICR mice at 40 days post infection (dpi) by the standard HCl-pepsin digestion method (Li et al., 2010). Three-day adult worms (AD3) and six-day adult worms (AD6) were recovered from the intestines of infected mice at 3 days post infection (dpi) and 6 dpi, respectively, as previously described. Newborn larvae (NBL) were collected from 6 dpi female adults cultured for 24 h at 37°C in RPMI-1640 media. Somatic extracts of AD6, AD3, NBL, and ML and ML excretory/secretory products (ES) were prepared as previously described (Wang et al., 2014; Yang et al., 2015). The collected parasites from the different development stages of *T. spiralis were* homogenized and then frozen in liquid nitrogen, which was repeated three times. Then, the homogenate was centrifuged at 10,000 g at 4°C for 1 h. In order to collect ES of ML, ML were incubated in pre-warmed serum-free RPMI 1640 medium at 37°C under 5% atmospheric CO_2_ for 18 h. After incubation, the supernatant was collected and concentrated.

### Sequence Analysis of *Ts*-FBPA

The complete coding sequence of *Ts*-FBPA (GenBank Accession No. XM_003374234) was initially obtained from GenBank at the National Center for Biotechnology Information (http://www.ncbi.nlm.nih.gov/).

The sequence characteristics of *Ts*-FBPA were analyzed on the Expasy website (http:// www.expasy.org/). The amino acid sequences of FBPA homologs from other organisms were aligned using Clustal W. Phylogenetic trees based on *Ts*-FBPA homologs were constructed using MEGA 7.0 with the neighbor-joining (NJ) method.

### Preparation of Recombinant *Ts*-FBPA (r*Ts*-FBPA)

Specific primers were synthesized according to the coding cDNA sequence of *Ts*-FBPA. The sequence encoding *Ts*-FBPA was amplified by PCR using primers carrying *Nde*I and *Xba*I restriction sites (forward 5′- CGC CA TATG atggccagtt attcgacatat−3′; reverse: 5′- GCTCTAGA tcagtatgcatgtccagctac 3′). After purification, PCR products were ligated into the pMD-19T vector (TaKaRa, China), followed by sub-cloning into the expression vector pCold I (TaKaRa, China) using T4 DNA ligase (New England BioLabs). The recombinant plasmid containing *Ts*-FBPA gene were transformed into *Escherichia coli* strain BL21 (DE3) for expression. The expression of the r*Ts*-FBPA protein was induced by the addition of 0.2 mM isopropyl β-D-1-thiogalactopyranoside (IPTG) for 16 h at 20°C. The r*Ts*-FBPA soluble protein was purified by Ni-affinity chromatography (Qiagen, Dusseldorf, Germany) according to the manufacturer's instructions. Recombinant protein was analyzed on 12% sodium dodecyl sulfate-polyacrylamide gel electrophoresis (SDS-PAGE) and the r*Ts*-FBPA concentration was measured using the BCA Protein Assay Kit (Thermo Fisher Scientific, Waltham, USA).

### Production of Mouse Polyclonal Antibody

For antisera production, three mice were immunized subcutaneously with 0.3 mg r*Ts*-FBPA mixed with Complete Freund′s Adjuvant. Booster immunizations were carried out at 2 weeks intervals with 0.2 mg r*Ts*-FBPA mixed with incomplete Freund′s Adjuvant. Serum samples were collected before each immunization and 1 week after the last immunization, and the antibody titers were determined by ELISA using the recombinant proteins as antigens. The serum samples were stored at −80°C.

### Transcriptional Analysis of *Ts*-FBPA at Different Developmental Stages

Transcription of *Ts*-FBPA at different developmental stages of *T. spiralis* was measured by reverse transcription quantiative PCR (qRCR) as previously (Liu et al., 2013). Trizol reagent (Invitrogen) was used to extract total RNA from AD3, AD6, NBL, and ML of *T. spiralis* according to the manufacturer's protocol, which was separately reverse-transcribed using Reverse Transcriptase (Promega, Southampton, UK).

The Ts-FBPA-specific primers was as follows: forward 5′- TTGCAAGCAACCGTATTGGC−3′ and reverse 5′- CACTGTATTTGCCTTGCGCC−3′. Individual samples were then PCR-amplified using Power SYBR green PCR Master Mix (Applied Biosystems, USA). Glyceraldehyde-3-phosphate dehydrogenase (GAPDH, GenBank accession No. AF452239) of *Trichinella* was selected as an internal control, and H_2_O was used as a negative control. The primers of GAPDH were designed as follows: forward, 5′- TTAATGTCGTGGCTGTGAAT-3′, and reverse, 5′- CCAGTAGAAG CAGGGATGAT−3′. Relative mRNA expression was analyzed according to the comparative 2^−ΔΔCT^ method.

### Western Blot Analysis

Crude antigens from ML, NBL, AD3, AD6, ES antigens of ML and r*Ts*-FBPA were, respectively, separated by 12% SDS-PAGE and transferred onto polyvinylidene difluoride (PVDF, Immobilon, Millipore, United States) (Li et al., 2015). After blocking in 5% skim milk in TBS containing 0.1% Tween 20(TBS-T) at 37°C for 1.5 h, the membrane was incubated at 37°C for 1 h with 1:500 diluted anti-r*Ts*-FBPA mouse sera and 1:200 diluted sera from pigs infected with 20,000 *T. spiralis* ML and collected at 60 dpi. Pre-immune mouse and pig serum served as a negative control. After washed five times with TBS-T, membranes were incubated with HRP-conjugated secondary antibodies (Sigma) diluted at 1:10,000 in TBST containing 5% skim milk for 1 h at room temperature. After washing the membranes five times again, the peroxidase activity was detected by the enhanced chemiluminescence (ECL) method.

### Immunofluorescence Assay (IFA)

The samples from different developmental stages of *T. spiralis* were fixed with 4% polyoxymethylene for 6 h and embedded in paraffin. The sections mounted on glass slides pre-coated with poly-L-lysine- were deparaffinized for 30 min in xylene and rehydrated. The slides were permeabilized with 0.1% Triton X-100 for 1 h, then treated with protease K (20 μg/ml) for 5 min. After washed three times with PBS, sections were blocked with 5% sheep serum for 30 min to reduce non-specific binding and then incubated overnight at 4°C with a 1:200 dilution of mouse anti-r*Ts*-FBPA serum. After washing three times again, sections were incubated with Alexa Fluor488 goat anti-mouse IgG for 1 h (Invitrogen) diluted at 1:100 in PBS for 1 h at room temperature. Subsequently, sections were incubated with 1 μg/ml Hoechst 33342 for 5 min at room temperature and then washed three times with PBS. sections were then mounted on glass slides in ProLong Gold antifade reagent (Invitrogen) and observed under laser scanning confocal microscopy (Fluoview FV 1000, Japan). The pre-immune serum from mice and the serum from mice infected with 300 *T. spiralis* ML collected at 40 dpi served as a negative and positive control, respectively.

### Immunization and Challenge Experiments

To determine the protective potential of the r*Ts*-FBPA protein, 75 female BALB/c mice were randomly divided into three groups of 25 mice each. The first group of mice was immunized subcutaneously with 30 μg of r*Ts*-FBPA emulsified in ISA206, then booster immunization was performed using the same method at 2 weeks intervals. The mice in the second and third groups were immunized with PBS emulsified with ISA206 or PBS only, as controls. One week after the final boost, spleens were collected to determine cellular immune responses. Two weeks after the final boost, all the mice were challenged orally with 300 *T. spiralis* ML. The reduction rate of adult worm or muscle larvae recovered from the vaccinated group was calculated compared with worms recovered from the PBS control mice.

### Analysis of Specific Antibody Responses by Indirect ELISA

Specific antibodies against *Ts*-FBPA were assayed at 6 weeks post vaccination (wpv). Blood was collected from vaccinated mice at 2, 4, and 6 wpv. The titers of anti-r*Ts*-FBPA IgG, IgG1, IgG2a subclasses, and IgE was measured using an indirect enzyme-linked immunosorbent assay (ELISA) as described previously (Feng et al., 2013).

### Cytokine Assays

In order to analyze the cellular immune responses of mice immunized with *Ts*-FBPA, the cytokine profile from splenocyte culture supernatants was measured as described previously (Feng et al., 2013). Briefly, 1 week after the last immunization, splenocyte suspensions were prepared from each group of mice and 2 × 10^6^ cells were cultured in RPMI 1640 with 10% fetal bovine serum (Sigma), 100 U/ml penicillin and 100 μg/ml streptomycin in a 24 well cell culture plate. After stimulation with 20 μg/ml r*Ts*-FBPA for 48 h at 37°C, the supernatants were collected and the concentration of cytokines including IL-2, IFN-γ, IL-4, and IL-10 was determined by ELISA assay according to the manufacturer's instructions (eBioscience, USA). Three replicates were tested for each sample.

### Statistical Analysis

Statistical analysis was performed using GraphPad Prism 5 (GraphPad InStatt Software, San Diego, California). Comparisons among the groups were performed with one-way or two-way analysis of variance (ANOVA). Data were displayed as the mean ± standard deviation (SD). *p*-values were expressed as ^*^p < 0.05, ^**^p < 0.01, and ^***^p < 0.001.

## Results

### Bioinformatic Analysis of *Ts*-FBPA Gene Sequences

**Figure 1:**
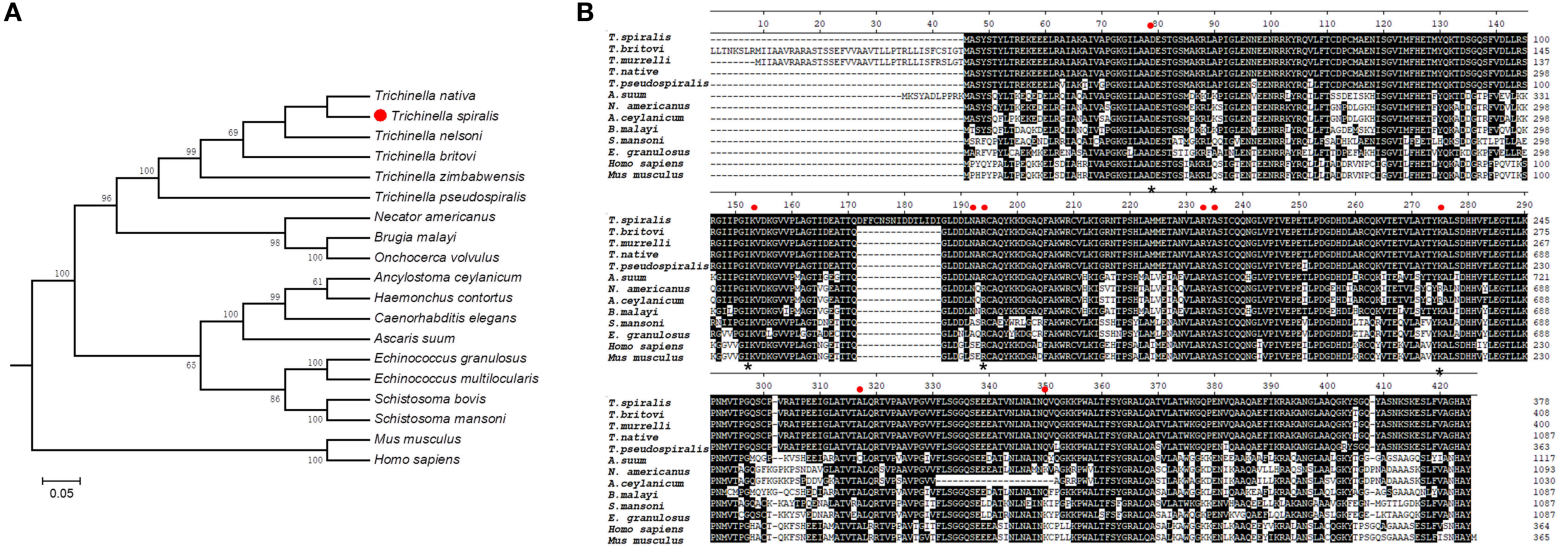
Sequence alignment and phylogenetic tree. **(A)** Multiple sequence alignment of FBPAs from *Trichinella spiralis* and homologs from other *Trichinella* species and other organisms. Active site residues are indicated in the red dot. Asterisks represent actin binding residues. Black shading and dashes represent the amino acid identity and gaps, respectively. **(B)** A phylogenetic tree of *Ts*-FBPAs and homologs from other species.

Bioinformatic analysis showed that *Ts*-FBPA belongs to class-I enzymes and the complete *Ts*-FBPA cDNA sequence was 1,137 bp which encodes 378 amino acids with a predicted molecular weight and isoelectric point of 41.1 kDa and 6.90, respectively. Comparing of sequence similarity using the deduced amino acid sequence indicated that *Ts*-FBPA had the highest homology (90~96% identity) to aldolase from the related organisms *T. nativa*. Furthermore, comparison of *Ts*-FBPA amino acid sequences with other species showed that the catalytic residues (Asp-34, Lys-108, Asn-147, Arg-149, Arg-188, Ala-190, Lys-230, Ala- 272, and Gln304) are not entirely conserved, but the F-actin binding residues (Asp-33, Arg-42, Lys-107, Arg-148, and Lys-229) are highly conserved (Figure 1A). Phylogenetic analysis of *Ts*-FBPA homologs is shown in Figure 1B. The results revealed that *Ts*-FBPA is genetically relevant to *Ts*-FBPA from *T. nativa* and *T. nelsoni*, and is much closer to nematode FBPAs than vertebrate FBPAs.

### Cloning, Expression, Purification of r*Ts*-FBPA

**Figure 2:**
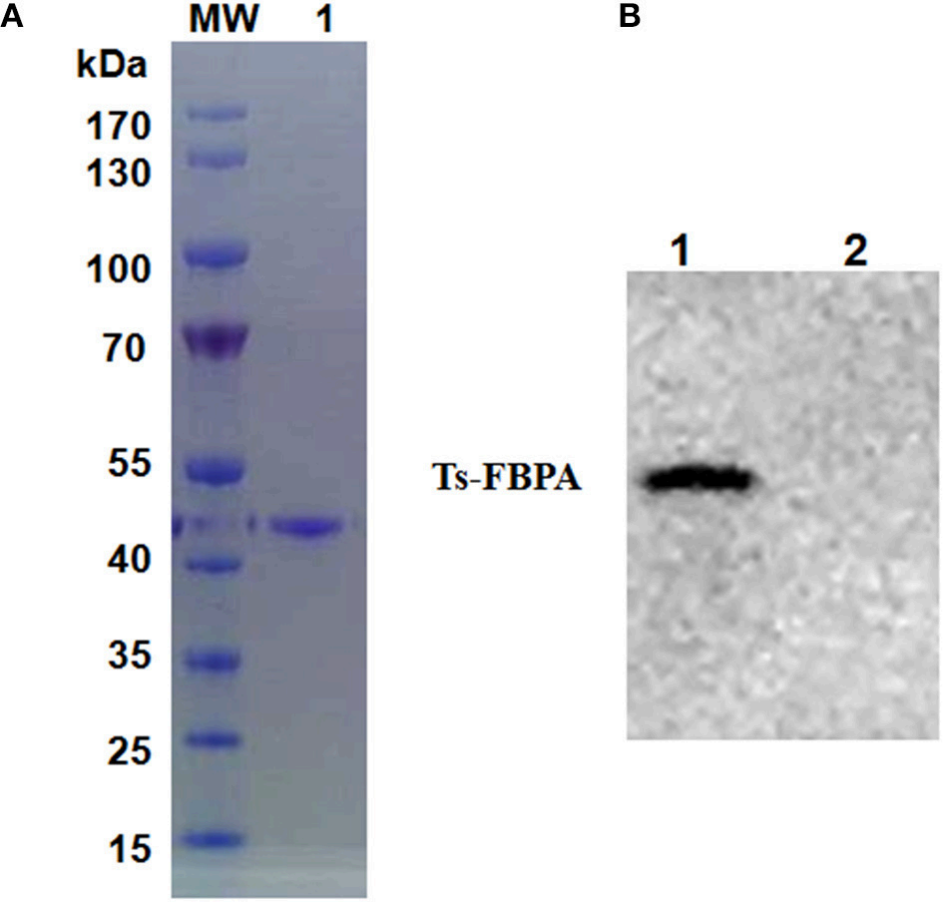
Purification and identification of recombinant *Ts*-FBPA. **(A)** Purified r*Ts*-FBPA was analyzed by 12% SDS-PAGE and stained with Coomassie Brilliant blue R. Lane MW: protein molecular weight marker, lane 1: purified r*Ts*-FBPA by Ni-affinity chromatograph. **(B)** The antigenicity of the r*Ts*-FBPA was analyzed by Western blotting. Lane 1: r*Ts*-FBPA incubated with sera from pigs infected with 20,000 ML of *T. spiralis* and collected at 60 dpi, Lane 2: r*Ts*-FBPA incubated with the sera from *Trichinella*–negative pigs.

According to the cDNA sequence of *Ts*-FBPA (GenBank Accession No. XM_003374234.1), specific primers were synthesized to amplify *Ts*-FBPA coding DNA. The amplified 1,137 bp of DNA was subcloned into the *E. coli* expression vector pCold I (TaKaRa, China). Soluble r*Ts*-FBPA with a His-tag at the N terminus was expressed in *E. coli* BL21 (DE3) with a size of approximately 42 kDa (Figure 2A) corresponding to the predicted molecular mass containing the His tag. Western blot showed that r*Ts*-FBPA was recognized by the *T. spiralis*-infected pig serum (Figure 2B, line 1). No recognition of *Ts*-FBPA was observed with the *Trichinella*-negative pig serum (Figure 2B, line 2). This result indicates that the FBPA of *T. spiralis* is an immunodominant antigen and the recombinant protein expressed in *E. coli* was antigenic.

### *Ts*-FBPA Is Expressed in Different Life Stages of *T. spirails*

**Figure 3:**
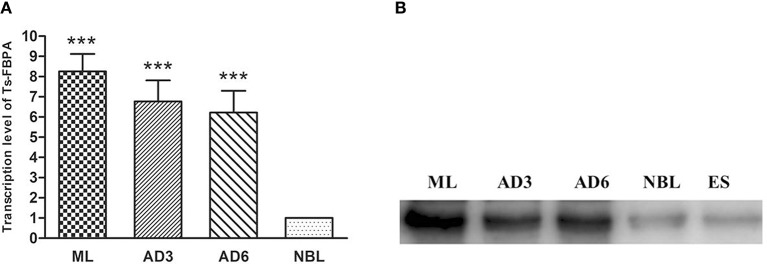
Analysis of the expression pattern of *Ts*-FBPA at different developmental stages of *T. spiralis*. **(A)** Revervse transcription qPCR analysis of the expression of *Ts*-FBPA at different developmental stages of *T. spiralis*. After reverse transcription, the cDNA of ML, AD3, AD6, and NBL was used as a template for qPCR using SYBR Green. Each reaction was performed in triplicate. All fold changes were relative to the NBL stage. Significant differences are as follows: ^***^P < 0.001. **(B)** Western blot analyses of Ts-FBPA expression levels at different developmental stages of *T. spiralis*. Expression levels of Ts-FBPA protein (41 kDa) in a crude antigen preparation of diverse *T. spiralis* growth phases were determined by Western blotting with 1:500 dilutions of anti-r*Ts*-FBPA serum. Line ML: *T. spiralis* ML crude parasite antigen, Line AD3: *T. spiralis* AD3 crude parasite antigen, Line AD6: *T. spiralis* AD6 crude parasite antigen, Line NBL: *T. spiralis*-NBL crude parasite antigen, Line ES: *T. spiralis*-ML ESPs.

The transcriptional profile of the *Ts*-FBPA gene at different developmental stages of *T. spiralis* was measured using qPCR with the transcription of GAPDH gene as a control. The results showed that transcription of the *Ts*-FBPA mRNA was detected at all developmental stages of *T. spiralis*. *Ts*-FBPA mRNA levels in NBL were lower than those in adult worms and ML. Compared with NBL, the mRNA expression level of *Ts*-FBPA was 8.9- and 5.6- fold higher in the ML and AD6, respectively. There were no statistically significant differences in *Ts*-FBPA expression levels between AD3 and AD6 (Figure 3A).

**Figure 4:**
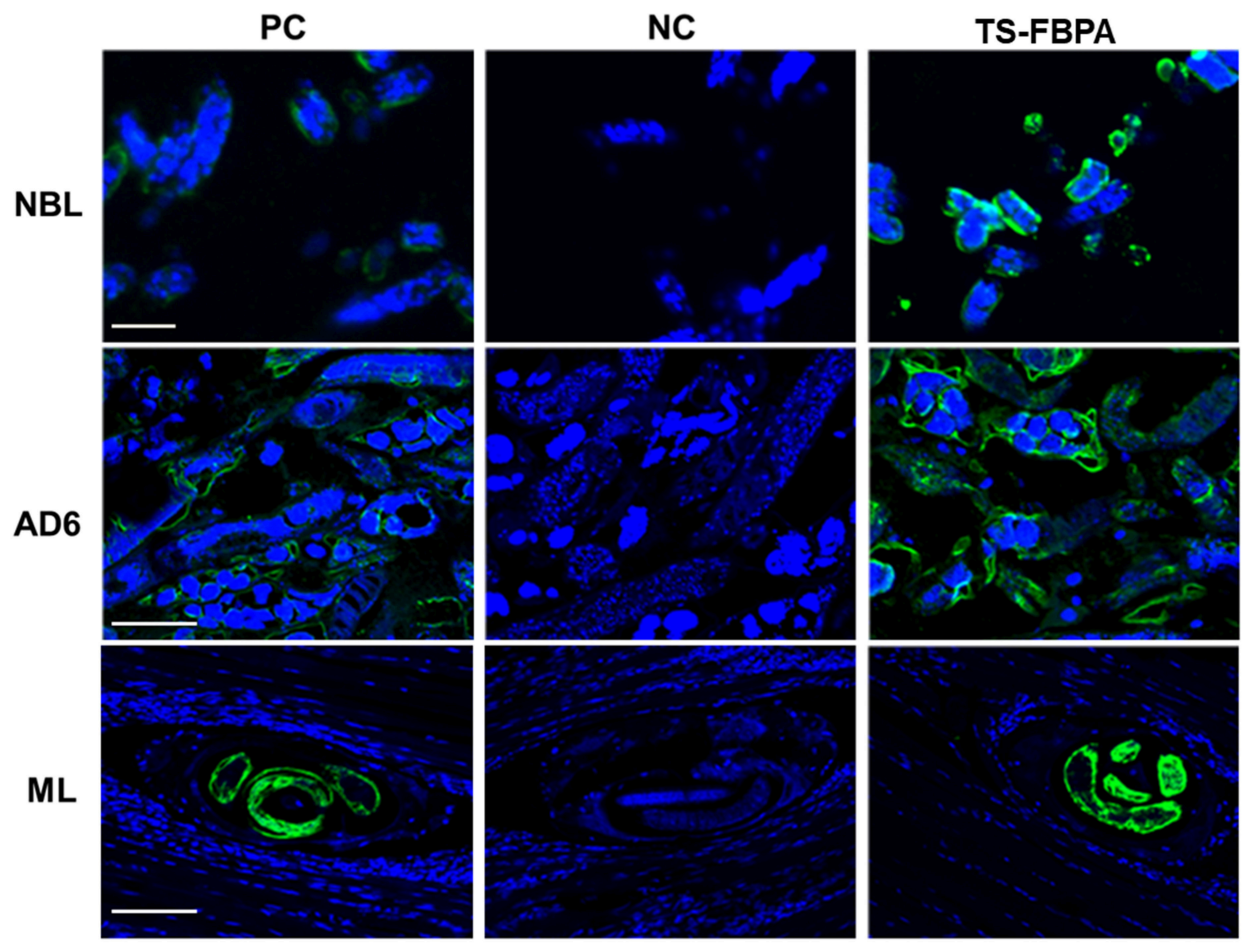
Immunolocalization of *Ts*-FBPA at different developmental stages of *T. spiralis*. sections of intact worms (NBL and AD) and skeletal muscles of infected mice (ML) were examined by IFA with anti-r*Ts*-FBPA sera. The obvious green fluorescent staining is observed at the cuticle of the parasites, especially on embryos within the female uterus. NC: pre-immune sera, PC: infection sera. Scale-bars: NBL 10 μm; AD6 and ML 30 μm.

To determine the expression of native *Ts*-FBPA in *T. spirails*, antigens from different development stages of *T. spiralis* were probed by western blot analysis using mouse polyclonal antibodies against r*Ts*-FBPA. The results showed that anti-r*Ts*-FBPA mice sera specially recognized native *Ts*-FBPA from various stages of *T. spiralis* including adult worms, ML and NBL. Furthermore, ES products from muscle larvae were recognized by the anti-r*Ts*-FBPA mice serum (Figure 3B), indicating that *Ts*-FBPA is a component of the ES antigens. To determine the distribution of *Ts*-FBPA in *T. spiralis*, IFA was performed using mouse anti-*Ts*-FBPA serum. The results showed that positive staining was found at the cuticles of AD and NBL and the embryos within the uterus of female adults at 6 dpi in the worm sections. When the sections of muscles from infected mice were treated with the anti-*Ts*-FBPA serum, positive staining was visible at the cuticle and internal organs of ML at 40 dpi (Figure 4). No immunostaining was seen in sections that were probed with pre-immune serum.

### Immune Protection of r*Ts*-FBPA Against *T. spiralis* Challenge

**Figure 5:**
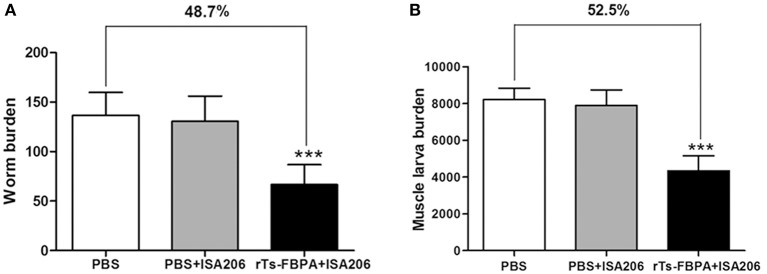
Protective immunity induced by immunizing mice with r*Ts*-FBPA against *T. spiralis* larval challenge. The number of adults recovered from intestines **(A)** and muscle larvae per gram (LPG) in skeletal muscles. **(B)** from immunized mice after challenge with 300 ML of *T. spiralis*. Results are expressed as the mean ± SD of 10 mice per group. Asterisks indicate significant differences (^***^P < 0.001) in adults/larvae recovered from the vaccinated group compared to PBS control groups.

The protective efficacy of *Ts*-FBPA against *T. spirails* infection was investigated in immunized mice. Compared with mice in the PBS-immunized group, mice in the r*Ts*-FBPA-immunized group exhibited 48.7 and 52.5% reduction of worm burden in adult worm (Figure 5A) and muscle larvae, respectively, (Figure 5B) after challenge with 300 *T. spiralis* infectious larvae. No significant difference was found in adult and larvae burden between mice immunized with adjuvant and PBS. This result demonstrated the immune response triggered by r*Ts*-FBPA induced partial protection against challenge with *T. spiralis* larvae.

### Specific Immunoglobulin Responses and Cytokines in Vaccinated Mice

**Figure 6:**
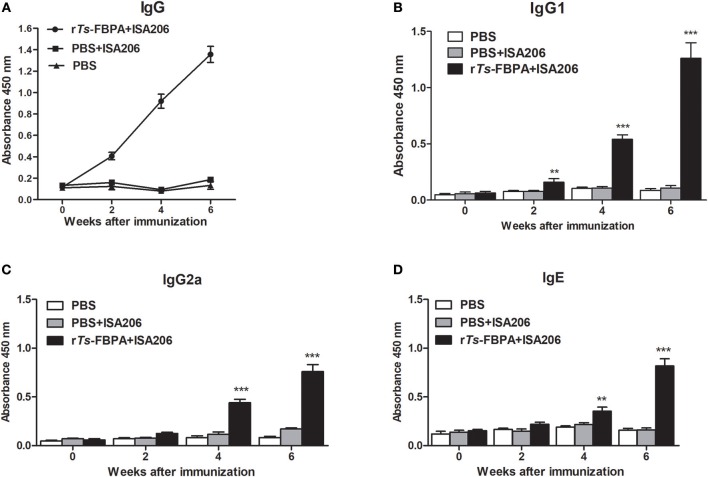
Analysis of humoral immune responses to r*Ts*-FBPA. **(A)** The levels of anti-r*Ts*-FPBA IgG in the sera of immunized mice or control (ISA006 or PBS) mice were measured by ELISA. **(B)** The IgG1 subclass responses against r*Ts*-FBPA were detected at different time points. **(C)** The IgG2a subclass responses against r*Ts*-FBPA were detected at different time points. **(D)** The levels of specific IgE r*Ts*-FBPA in the sera of immunized mice were measured. The values shown for each group are the mean + SD of the antibody levels (*n* = 10). Significant differences were as follows: ^**^P < 0.01, ^***^P < 0.001.

To determine humoral antibody responses to r*Ts*-FBPA in immunized mice, the serum samples were collected 2 weeks after each immunization, then the titers of specific IgG and its subclasses (IgG1 and IgG2a) antibodies against r*Ts*-FBPA were assayed by ELISA. The results showed significant increase in total serum IgG levels in mice immunized with r*Ts*-FBPA compared with the control and continued to rise after the third immunization (Figure 6A).

The IgG subtype assay indicated that IgG1 and IgG2a responses were both induced after mice immunized with r*Ts*-FBPA and their expression levels were significantly upregulated upon boost vaccinations (Figures 6B,C). The IgG1 level was higher than IgG2a, indicating that immunization with r*Ts*-FBPA induced IgG1-dominant Th1(IgG2a)/Th2(IgG1) -mixed type immune response. The levels of specific IgE were also investigated and the results revealed that its levels were markedly increased in mice immunized with r*Ts*-FBPA compared with the control (Figure 6D), indicating that IgE antibodies could play important roles in *Ts*-FBPA induced rapid expulsion of intestinal worms from the intestine.

**Figure 7:**
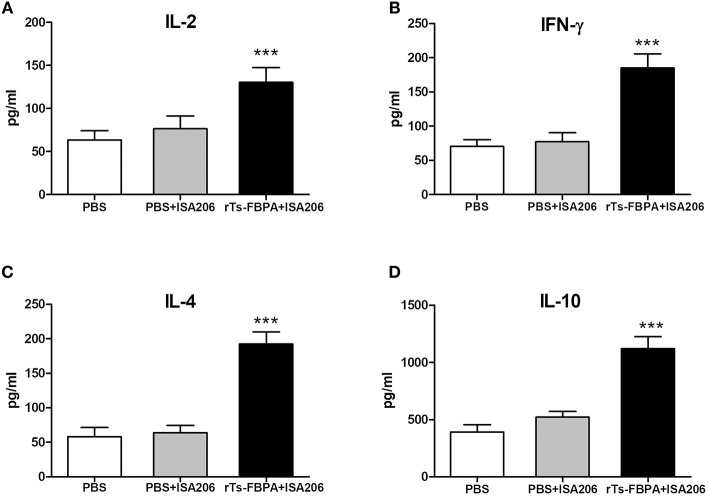
Analysis of cytokine production from splenocytes after rTs-FBPA stimulation *in vitro*. Splenocytes secreting IL-2, IFN-γ, IL-4 and IL-10 were detected by ELISA one week after the final immunization. The values of **(A)** IL-2, **(B)** IFN-γ, **(C)** IL-4 and **(D)** IL-10 are presented as the mean ± SD of 5 mice per group. The asterisks (^*^) indicate that the production of cytokines by immunized mice was significantly different (^***^P < 0.001) from that of the PBS control group.

To further confirm that a Th1/Th2-mixed response was induced after vaccination with r*Ts*-FBPA, levels of Th1/Th2 cytokines, including in IL-2, IFN-γ, IL-4, and IL-10, were detected. Compared with the adjuvant and PBS control groups, obviously higher expression of IL-2, IFN-γ, IL-4, and IL-10 were found in the supernatants of r*Ts*-FBPA-stimulated splenocytes from mice immunized with r*Ts*-FBPA (Figure 7), confirming that a Th1/Th2-mixed immune response was significantly induced by immunization with r*Ts*-FBPA.

## Discussion

Enzymes associated with the glycolytic pathway that is used to generate energy in nematodes play important roles in parasite survival in the host. FBPA is an essential enzyme in energy production by carbohydrate metabolism and has been identified and characterized in some parasites (El-Dabaa et al., 1998; Starnes et al., 2009; Li et al., 2014). Beyond its normal glycolytic role, several studies have shown that it plays important roles in host invasion (Hu et al., 2015). The pleiotropic functions of FBPA in the biology of these parasites suggest it may be a promising target for developing novel nematocidal drugs and vaccines. In the present study, we firstly characterized the molecular characterization and expression of FBPA in *T. spiralis*, and evaluated its protective effect in mice.

Amino acid sequence comparisons showed that the catalytic residues were not entirely conserved. The diversity of some active sites might result from different parasite environments among these species during evolution. The influence of changes in the catalytic residues in the schiff base binding site formation and enzyme-catalyzed activity is unknown, and this will be investigated in a future study. Interestingly, highly conserved amino acid residues related to actin binding were present in the *Ts*-FBPA, suggesting *Ts*-FBPA could have non-glycolytic functions. In apicomplexan parasites, FBPA plays an important role in bridging adhesin-cytoskeleton interactions, which facilitate parasite motility and host invasion. However, the role of the actin binding site identified in *Ts*-FBPA needs further investigation.

The characteristics of *Ts*-FBPA were determined at mRNA and protein levels by qPCR, western blotting and IFA. The qPCR and Western Blot results showed that *Ts*-FBPA was expressed during various developmental stages of *T. spiralis* (NBL, ML, AD3, and AD6), suggesting essential roles of *Ts*-FBPA in glycolysis throughout the life stages. Although *Ts*-FBPA was expressed in all development stages of *T. spiralis*, its transcription levels in ML and adult worms were significantly higher than that in NBL, and there was no statistically significant difference between the AD3 and AD6 stages, which is consistent with the high energy requirement for growth and development during these stages. A previous study has indicated that fructose-1,6-diphosphate was required for maximum activity in phosphorylative glycolysis in the ML of *T. spiralis* (Agosin and Aravena, 1959).

Generally, these extracellular locations of protein are considered to be involved in moonlighting functions (de la Paz Santangelo et al., 2011). Some studies have indicated that FBPA was expressed on the parasite surface to exert functions associated with parasite motility and invasion by connecting surface-adhesive proteins to the actin–myosin motor of the parasite (Diaz et al., 2014). In *Mycoplasma bovis*, FBPA localizes to the surface of the cell membrane and binds plasminogen, where it utilizes the activity of hydrolytic surface-associated proteins to help invasion of host cell (Gao et al., 2018). Our studies found that the *Ts*-FBPA expressed at parasite surface and secreted out of the cell facilitated the interaction with host and might play a pivotal non-glycolytic role in various micro-environments. The moonlighting functions of *Ts*-FBPA require further investigation.

Considering the role of *Ts*-FBPA in glycometabolism and its expression at all growth stages of *T. spiralis*, as well as its exposure on the surface of the parasite that have been indicated to be pivotal in conferring protection against *T. spiralis* infection, mice were immunized with r*Ts*-FBPA to evaluate the protective efficacy of this recombinant protein. The results showed that immunization of mice with r*Ts*-FBPA induced 51.8 and 48.6% reduction of worm burden in adult worm and muscle larvae, respectively, compared to control mice injected with PBS. Similarly, FBPAs in other helminths such as *Onchocerca volvulus* (McCarthy et al., 2002) and *Schistosoma mansoni* (Marques et al., 2008) also were found to provide significant protection against parasite infection in experimentally infected animals. Furthermore, the r*Sj-*FBPA protein showed promising diagnostic potential in *Schistosoma japonicum* infected water-buffaloes and with 100% sensitivity and specificity in a detection assay (Peng et al., 2008). These results indicated that r*Ts*-FBPA may have the potential to inhibit parasite invasion and development and may be a vaccine candidate.

To further investigate the mechanism underlying the protective immunity induced by r*Ts*-FBPA, we analyzed humoral and cell-mediated immune responses induced by r*Ts*-FBPA. A previous study indicated that protective immunity against helminth infection is strongly associated with helminth-specific antibodies (McCoy et al., 2008). The passive transfer of this antibody against different immunodominant antigens to naive mice can induce significant protection against *T. spiralis* larval infection (Bell et al., 1992). Our results showed that r*Ts*-FBPA emulsified in ISA206 adjuvant triggered a mixed IgG1/IgG2a antibody response, with IgG1 being predominant. This result is in agreement with previous studies that reported that combined Th1 and Th2 immune responses induced by recombinant proteins were important for substantial protection against *T. spiralis* infection (Feng et al., 2013; Bi et al., 2015; Yang et al., 2018). IgG1 antibodies efficiently bind to Fc gamma receptors (FcγRs) on effector cells and play a key role in antibody-dependent cell-mediated cytotoxicity to protect against a large multi-cellular parasite. Previous studies have indicated that anti-*Trichinella* specific antibodies promote the death of NBL and ML through antibody-dependent cell-mediated cytotoxicity (ADCC) and the activation of complement (Sun et al., 2018). A recent study found that helminth-specific IgG1 co-operates with effector cells to rapidly trap tissue migrating helminth larvae and prevent tissue necrosis following infection with *Heligmosomoides polygyrus bakeri* (Hp) (Esser-von Bieren et al., 2013). Our study indicated that the induction of specific antibodies against r*Ts*-FBPA may be the essential components in diminishing the effect of *T. spiralis* challenge. Furthermore, we also analyzed the expression of IgE, which exerts a key function in rapidly expelling adult worms from the intestine by mediating mast cell degranulation (Gurish et al., 2004). In addition, IgE also plays a critical role in killing NBL by cytotoxic attack (Falduto et al., 2015). Here, our results indicated that immunization with r*Ts*-FBPA induced a high level of specific anti-*Ts*-FBPA IgE antibodies in mice, which was coincident with the reduced burden of parasites in the intestine and muscle of mice immunized with r*Ts*-FBPA. Thus, these results suggest that IgE may be involved in the protective immune response against *T. spiralis* infection. Further studies are required to better understand the underlying immune mechanisms by which antibodies act against these multicellular parasites.

Although a strong antibody response was important for protective immunity, it was not sufficient and cellular immunity was also required for elimination of the helminth parasite. In our study, cytokines of IL-2, IFN-γ, IL-4, and IL-10 secreted by the splenocytes of mice immunized with r*Ts*-FBPA were significantly elevated. The production of these cytokines confirms that r*Ts*-FBPA induced a Th1/Th2 mixed immune response. It is well-known that expulsion of *T. spiralis* is related to prominent mastocytosis mediated by a Th2-type response involving IL-4 (Urban et al., 2000). IL-4R alpha-deficient mice exhibit substantially reduced parasite expulsion, intestinal pathology and Th2 responses (Scales et al., 2007). In this regard, IL-4 is a critical mediator of the humoral immune response. Despite high levels of pro-inflammatory cytokines were induced, abundant IL-10 were also released in the vaccinated groups. IL-10 has a dual role in protective immune responses directed against the diverse life stages of *T. spiralis* parasites. IL-4 plays an important role as a positive regulator of intestinal mast cell responses and is involved in expelling intestinal adult worms *in vivo* (Ding et al., 2017). On the contrary, IL-10 has a negative effect on protecting newborn larvae, and the lack of IL-10 leads to improved immunity against NBL, as well as decreased numbers of ML cysts, which is associated with elevated IFN-γ (Helmby and Grencis, 2003). IFN-γ can enhance cytotoxic killing by eosinophils, granulocytes and activated macrophages to exert its protective effects against NBL. The balance between IFN-γ and IL-10 is crucial in determining protective immunity against different phases of *T. spiralis*. Given the important roles for these cytokines in immunity against parasite infection, our results confirmed that FBPA can elicit protective immunity and is a potential vaccine candidate.

The route of immunization can influence the degree of success in conferring protective immunity (Li et al., 2018). *T. spiralis* infection occurs in the same host with two distinct intracellular niches: the intestinal epithelium and the skeletal muscle cells. During the intestinal phase, intestinal infective larvae (IIL) invade intestinal epithelia where they molt to adult worm, mate and produce. Then, female worms release newborn larvae (NBL) that penetrate into the intestinal wall and migrate to skeletal muscles through the bloodstream and lymphatic circulation and develop muscle larvae. Obviously, the first natural site of *T. spiralis* infection is the mucosal surface of the intestine, and local mucosal immunity in the gut that guard against parasite invasion and mucosal colonization could be crucial in protection against intestinal *Trichinella* infection. The oral immunization with live attenuated Salmonella-delivered *Trichinella* DNA vaccines as an interesting alternative vaccination route that has been proven to have potential in inducing both systemic and mucosal immune responses which provides high protection against *T. spiralis* infection (Liu et al., 2015; Wang et al., 2016; Li et al., 2018; Qi et al., 2018). The protective efficacy in oral vaccination of mice with attenuated Salmonella expressing FBPA antigen will be further investigate.

In conclusion, our study indicated that *Ts*-FBPA was ubiquitously expressed at each developmental stage of *T. spiralis* and the protein was located at cuticles of the parasite and embryos, suggesting that the *Ts*-FBPA plays an important role in invasion and survival. Further functional experiments are required to elucidate the physiological and biological role of FBPA in parasite metabolism itself and *Trichinella*-host interaction. In addition, our study also indicated that mice immunized with r*Ts*-FBPA had partial protective immunity against *T. spiralis* infection, however, we have yet to perform a clear picture of how immunity acts against infection with parasites. The mechanism by which *Ts*-FBPA induces a protective immune response against *T. spiralis* infection requires further investigation.

## Data Availability

All datasets generated for this study are included in the manuscript and/or the supplementary files.

## Ethics Statement

This study was carried out in accordance with the recommendations of the Administration of Affairs Concerning Experimental Animals in China. The protocol was approved by the Institutional Animal Care and Use Committee of Jilin University (20170318).

## Author Contributions

The study was conceived and designed by ML and XL. YY, CL, and MT performed the experiments. YY, XB, and CL analyzed the data. YY and XB wrote the manuscript. PZ and WC improved the manuscript. All authors read and approved the final manuscript.

### Conflict of Interest Statement

The authors declare that the research was conducted in the absence of any commercial or financial relationships that could be construed as a potential conflict of interest.
